# The clinical and pathological histology efficacy of biological therapy for severe asthma with a phenotype of type 2 inflammation - systematic review

**DOI:** 10.3389/fimmu.2025.1531986

**Published:** 2025-04-15

**Authors:** Junhui Ma, Qiang Ma, Jing Yang, Panpan Liang, Jiaxin Zhou, Jiarui Ma, Fuhua Ma, Bing Zhuan, Wei Zhou

**Affiliations:** ^1^ Department of Respiratory Medicine, People's Hospital of Ningxia Hui Autonomous Region, Ningxia Medical University, Yinchuan, Ningxia, China; ^2^ Department of Chest Surgery, People’s Hospital of Ningxia Hui Autonomous Region, Yinchuan, China; ^3^ Medical Administration Department, Yongning County People’s Hospital, Yinchuan, China

**Keywords:** severe asthma, type 2 inflammation, biologic therapy, pathological histology efficacy, phenotype

## Abstract

Asthma is a complex, chronic inflammatory condition of the airways that comes in many forms. Because different inflammatory processes drive it, we can generally categorize asthma into two main types: type 2 inflammatory asthma and non-type 2 inflammatory asthma. Type 2 inflammation is usually the culprit in most folks grappling with severe asthma. There is a noticeable difference in the treatment approaches for different phenotypes of severe asthma. The main reason is that patients suffering from type 2 inflammatory asthma can respond well to treatment with biological agents. Several well-verified biological agents, such as anti-immunoglobulin E (IgE) monoclonal antibodies, anti-interleukin (IL)-4 monoclonal antibodies, anti-IL-5 monoclonal antibodies, and anti-thymic stromal lymphopoietin (TSLP) monoclonal antibodies, have shown outstanding effectiveness. They can significantly alleviate asthma exacerbations, lower the number of eosinophils, improve pulmonary function, decrease the dependence on oral corticosteroids, and elevate the quality of life for patients with asthma. This discourse meticulously evaluates the therapeutic prowess of biological agents in the treatment and control of severe asthma, concurrently investigating their impact on histological indices, to highlight the crucial role of precision medicine in the strategic concatenation of therapy for this refractory malady.

## 1 Introduction

Asthma ranks as the most prevalent inflammatory disorder affecting the pulmonary system (1). Afflicted by a mutable spectrum of respiratory symptoms, including dyspnea, persistent cough, and a sensation of thoracic constriction, asthma is linked to persistent inflammatory reactions within the airways, a reversible impediment to expiratory airflow, and heightened responsiveness of the bronchial passages (2). Asthma impacts more than 300 million people worldwide, with a significant segment—around 2.5% of minors and 10% of adults—experiencing its more severe forms. This can lead to serious complications such as persistent airflow restrictions, frequent flare-ups, hospitalizations, and even mortality, ultimately diminishing quality of life and increasing healthcare expenditures (2). Severe asthma refers to the condition that persists as uncontrolled even after implementing enhanced high-dose ICS-LABA treatment and tackling all contributing factors, or when the condition deteriorates upon reduction of high-dose treatment (according to the 2024 GINA report) (3). Consequently, it becomes apparent from the very definition that managing severe asthma constitutes a formidable endeavor.

Given the varied ways severe asthma manifests, pinpointing the right treatment approach is becoming more and more crucial (4). This means we need to be smart about weighing safety, how well a treatment works, and cost-effectiveness for each specific type of asthma. The arrival of cutting-edge biologics, alongside better biomarkers, has really paved the way for personalized treatments that hit the mark for those struggling with severe asthma (4, 5). Five biologics are approved for eosinophilic asthma management. A comprehensive review assessing the effectiveness and safety of these agents demonstrated that each one significantly lowers the incidence of severe asthma flare-ups. Additionally, it was noted that benralizumab, dupilumab, and mepolizumab also contribute to a reduced dependence on oral corticosteroids(OCS) (4, 6). Numerous investigations indicate biological therapies can improve asthma management, life quality, and pulmonary performance (7).

The hallmark pathological shifts in asthma stem from the intricate interplay and influx of immune cells, including eosinophils, neutrophils, lymphocytes, dendritic cells (DCs), mast cells and innate lymphoid cells (ILCs), which collectively fuel persistent inflammation in the airway walls (8). This inflammatory cascade triggers airway constriction, heightened bronchial hyperresponsiveness, mucus-induced blockages, and structural alterations in the airways (9). The long-term effectiveness of biological therapies for severe asthma should also be assessed based on their ability to improve these pathological changes. This paper reviews advances in research evaluating biological therapies’ histopathological efficacy in acute asthma.

## 2 Search strategy and study selection

The conduct and reporting of our research followed the PRISMA Extension for Scoping Reviews (PRISMA-ScR) guidelines (10).

### 2.1 Search strategy

#### 2.1.1 Databases searched

We scoured several electronic databases for relevant studies. Our search took in PubMed, Embase, and the Cochrane Central Register of Controlled Trials. We chose these databases because they offer broad coverage of the medical literature, including tons of peer-reviewed articles, clinical trials, and systematic reviews, particularly those dealing with respiratory illnesses like asthma.

#### 2.1.2 Search terms

The search terms were carefully formulated to capture all relevant studies on the clinical and pathological histology efficacy of biological therapy for severe asthma with a type 2 inflammation phenotype. The search strategy combined medical subject headings (MeSH) terms and free - text words.

MeSH Terms: “Asthma”, “Severe Asthma”, “Type 2 Inflammation”, “Biological Therapy”, “Clinical Efficacy”, “Pathological Histology”.

Free - text Words: “anti - IgE”, “anti - IL - 4”, “anti - IL - 5”, “anti - TSLP”, “dupilumab”, “mepolizumab”, “reslizumab”, “benralizumab”, along with their brand names. These terms were used to cover different biological agents commonly used in the treatment of asthma. Additionally, terms like “eosinophilic asthma”, “Th2 - high asthma”, “biomarkers of type 2 inflammation”, “lung function improvement”, “histological changes in asthma”, etc., were included to ensure a comprehensive search.

#### 2.1.3 Search syntax

In PubMed, the search syntax was constructed as follows: (“Asthma”[Mesh] AND “Severe Asthma”[tiab] AND “Type 2 Inflammation”[tiab]) OR (“Biological Therapy”[Mesh] AND “Clinical Efficacy”[tiab] AND “Pathological Histology”[tiab]) OR (“anti - IgE”[tiab] OR “omalizumab”[tiab] OR “anti - IL - 4”[tiab] OR “dupilumab”[tiab] OR “anti - IL - 5”[tiab] OR “mepolizumab”[tiab] OR “reslizumab”[tiab] OR “benralizumab”[tiab] OR “anti - TSLP”[tiab]) AND (“eosinophilic asthma”[tiab] OR “Th2 - high asthma”[tiab] OR “biomarkers of type 2 inflammation”[tiab] OR “lung function improvement”[tiab] OR “histological changes in asthma”[tiab]). The “tiab” operator was used to search in both the title and abstract of the articles.

In Embase, a similar search strategy was implemented, adjusting the indexing terms according to Embase’s thesaurus. The search was designed to retrieve all relevant articles published in the English language.

For the Cochrane Central Register of Controlled Trials, the search focused on clinical trials related to the topic. The search terms were adapted to fit the Cochrane database’s search interface, with a particular emphasis on identifying randomized controlled trials, which are considered the gold standard for evaluating treatment efficacy.

### 2.2 Study selection

#### 2.2.1 Inclusion criteria

Study Design: Only human studies were included. Randomized controlled trials (RCTs), cohort studies, and case - control studies were considered eligible. RCTs were given priority as they provide the most reliable evidence for treatment efficacy. However, cohort and case - control studies were also included to supplement the evidence, especially when RCTs were scarce in certain aspects.

Patient Population: Studies had to involve patients diagnosed with severe asthma. The diagnosis of severe asthma was based on international guidelines, such as those from the GINA. Additionally, the patients in the studies had to have a documented type 2 inflammation phenotype. This was determined by the presence of biomarkers such as elevated blood eosinophil counts, increased fractional exhaled nitric oxide (FeNO) levels, or positive immunohistochemical or histological evidence of type 2 - related cytokines and cells in the airway tissue.

Intervention: The studies had to evaluate the use of biological therapies for the treatment of severe asthma with a type 2 inflammation phenotype. Biological therapies included monoclonal antibodies targeting type 2 cytokines or other biological agents specifically designed to modulate the type 2 inflammatory pathway.

Outcome Measures: Studies reporting on either clinical efficacy outcomes (such as asthma exacerbation rate, lung function improvement, symptom control) or pathological histology outcomes (such as changes in airway inflammation, eosinophil infiltration, epithelial cell changes) were included. Studies that reported on both types of outcomes were particularly valuable.

#### 2.2.2 Exclusion criteria

Language: Articles not published in English were excluded. This was due to resource limitations, as the review team had the capacity to comprehensively review only English - language articles.

Study type: *In vitro* studies, animal studies, and case reports were excluded. While *in vitro* and animal studies can provide valuable insights into the mechanisms of action, they do not directly reflect the clinical and pathological effects in humans. Case reports, on the other hand, are often anecdotal and may not provide sufficient evidence for a systematic review.

Irrelevant Interventions or Outcomes: Studies that evaluated non - biological therapies for asthma, or those that did not report on the relevant clinical or pathological histology outcomes related to the type 2 inflammation phenotype in severe asthma, were excluded. For example, studies focusing solely on the use of bronchodilators or corticosteroids without any assessment of biological therapies were not included.

### 2.3 Selection process

Title and Abstract Screening: The initial results obtained from each database were downloaded and imported into a reference management tool like EndNote. All duplicate entries were eliminated. Two reviewers conducted an independent evaluation of the titles and abstracts of the remaining articles, using predefined inclusion and exclusion criteria. Any disagreements that arose were settled through discussion, and if needed, a third reviewer was brought in to help reach an agreement.

Full - text Review: For the articles that successfully passed the title and abstract screening, full texts were retrieved. The same two reviewers then independently assessed these documents to verify their eligibility. They extracted pertinent information from the qualifying articles, such as study design, patient demographics, intervention specifics, and outcome metrics. Any differences in data extraction were again handled through discussion or with assistance from a third reviewer when necessary.

## 3 Diagnosis and differentiation of severe asthma

**Figure 1:**
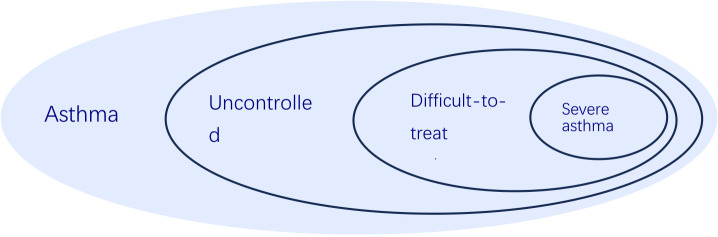
The relationship of uncontrolled asthma, difficult-to-treat asthma, severe asthma and asthma, according to 2024 GINA report.

**Figure 2:**
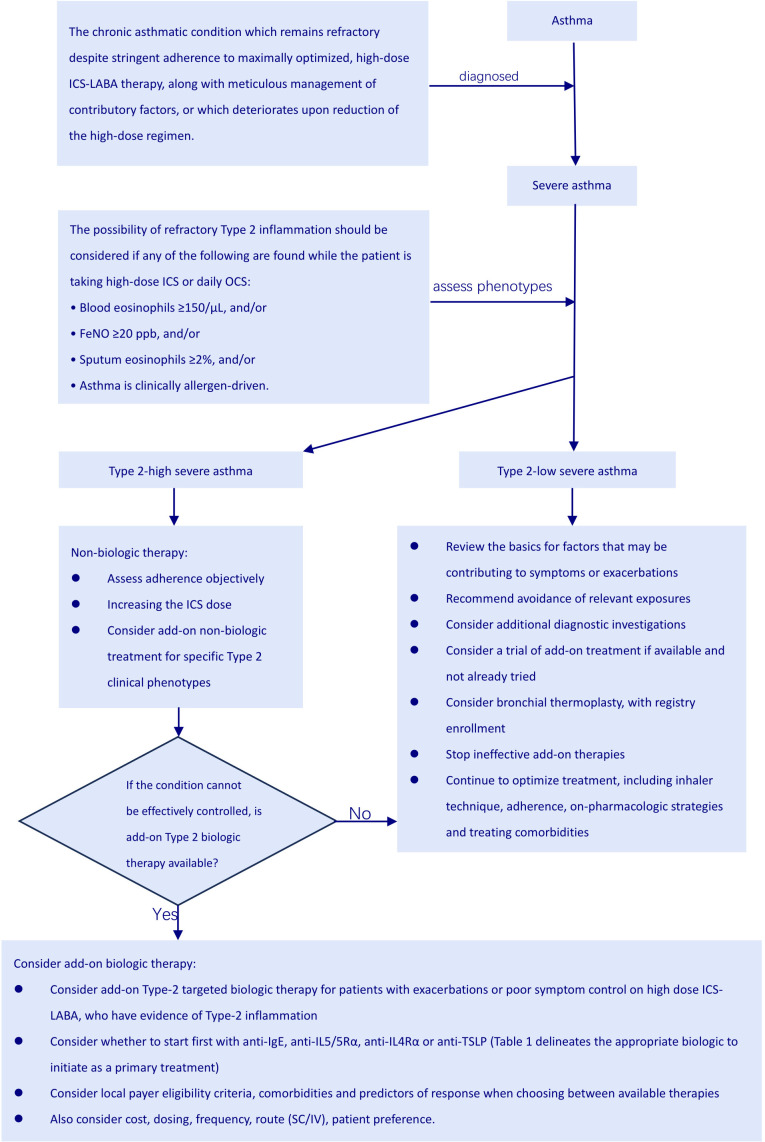
Severe asthma patients biological agent treatment flowchart.

Figuring out whether someone truly has severe asthma can be tricky because you must rule out cases where the asthma is simply poorly controlled or proving difficult to treat. Asthma is considered uncontrolled if someone is experiencing either of the following: (1) their symptoms aren’t well-managed – think frequent flare-ups, constantly needing their rescue inhaler, asthma keeping them from doing the things they enjoy, or asthma symptoms waking them up at night. (2) They’re having frequent asthma attacks – we’re talking two or more a year needing oral steroids, or at least one really bad attack a year that lands them in the hospital (3). When asthma proves stubborn and doesn’t respond to the usual treatment of combined medium-to-high dose inhaled corticosteroids (ICS) and long-acting beta-agonists (LABA), or when keeping symptoms at bay and flare-ups down necessitates hefty doses of ICS-LABA, we’re talking about difficult-to-treat asthma (3). Severe asthma is basically a tougher nut to crack within the larger scope of difficult-to-manage asthma (**Figure 1**). It describes a situation where asthma symptoms persist despite following a rigorous regimen of the highest recommended doses of ICS-LABA therapy and addressing all associated factors. Furthermore, this condition tends to worsen if the dosage of the treatment is lowered (**Figure 2**) (11).

## 4 Role of type 2 inflammation and cytokines in severe asthma

**Figure 3:**
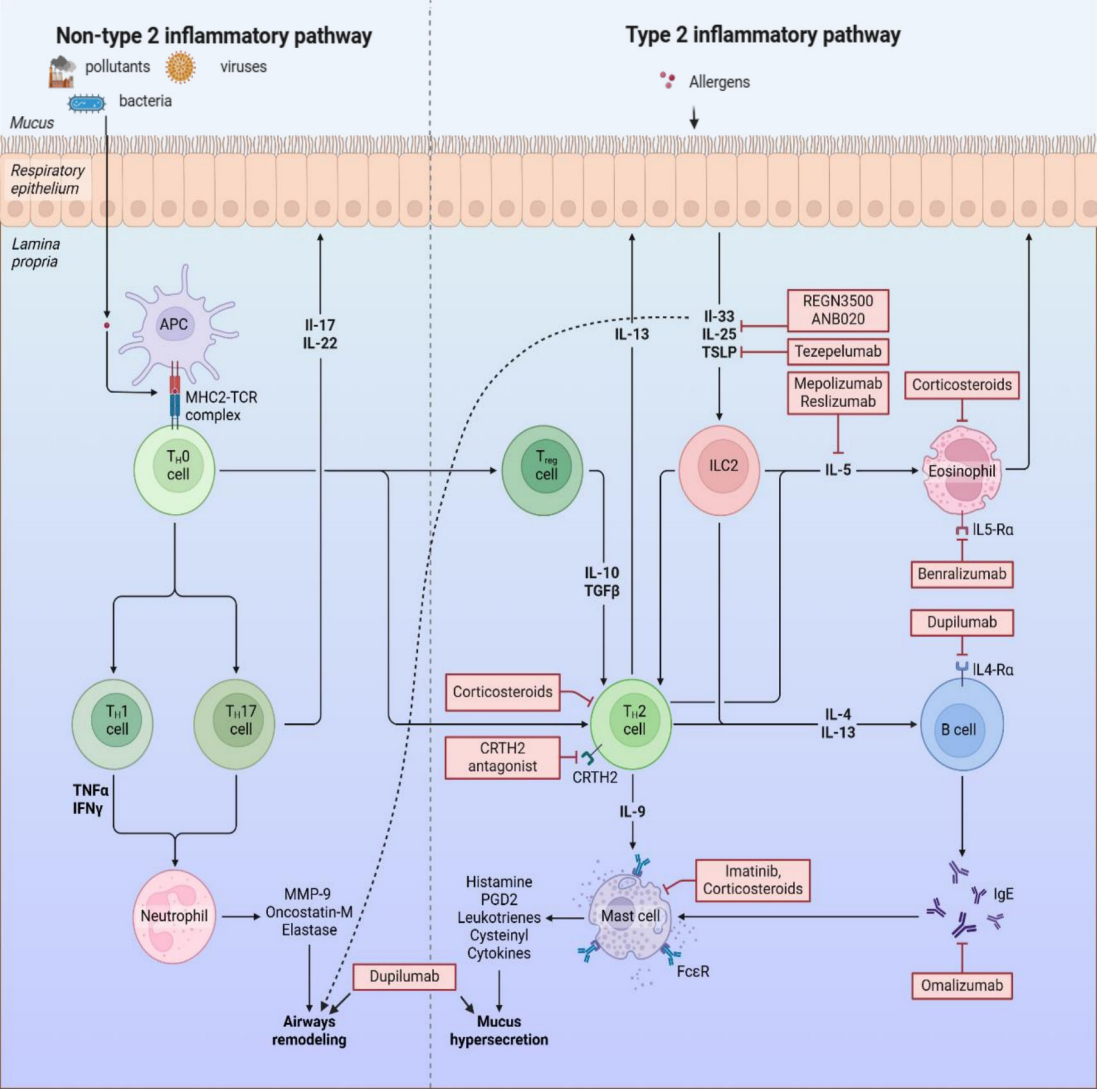
Airway inflammation and biological treatment targets in severe asthma. The 2024 GINA report categorizes individuals with severe asthma into two distinct biotypes: Type 2 inflammation and non-Type 2 inflammation. Type 2 inflammation: Due to the stimulation of allergens, pollutants, or microbes, bronchial epithelial cells (BEC) release IL-33, IL-25, and TSLP, which in turn activate group 2 innate lymphoid cells (ILC2) in the bronchial mucosa. Then, ILC2 and Th2 cell release IL-4, IL-5 and IL-13. The release of IL-5 is strongly correlated to eosinophilia by acting mainly on cells of eosinophilic origin, wherein it stimulates their growth and differentiation, enhances their survival and promotes the release of their granular contents at the site of inflammation. The IL-4 and IL-13 act together on B cells to induce the production of IgE. Th2 cells also release IL-9 to further activate mast cells, causing them to release histamine, PGD2, leukotrienes, cysteinyl, and cytokines, which cause mucus hypersecretion. The non-Type 2 inflammatory pathway is mainly mediated by neutrophils. When allergens are taken up by antigen-presenting cells (APC), they induce the differentiation of Tho cells into Th1 and Th17 cells, which in turn promote the release of MMP-9, elastase, and onconstain-M from neutrophils, leading to airway remodeling.

According to the 2024 GINA report, type 2 inflammation is a dominant factor in the most of severe asthma cases. When bronchial epithelial cells (BEC) encounter allergens, they respond by releasing a variety of cytokines, including IL-33, IL-25, and TSLP. These cytokines act as critical signaling molecules that subsequently activate type 2 innate lymphoid cells (ILC2) residing within the bronchial mucosa. The activation of ILC2 cells then triggers a cascade of downstream effects, further amplifying the type 2 inflammatory response and contributing to the chronic airway inflammation characteristic of severe asthma. This intricate interplay between epithelial cells and immune cells highlights the pivotal role of the airway epithelium in initiating and perpetuating type 2 inflammation in the context of severe asthma (12, 13). Subsequently, ILC2 and Th2 cells produce IL-4, IL-5, and IL-13. Notably, the secretion of IL-5 is closely linked to eosinophilia, as it primarily influences eosinophil progenitor cells, driving their growth and differentiation, enhancing their longevity, and promoting the release of their granular components at inflammation sites. Meanwhile, IL-4 and IL-13 work in concert to stimulate B cells to generate IgE. Additionally, Th2 cells produce IL-9, which further activates mast cells, leading them to release histamine, PGD2, leukotrienes, cysteinyl, and cytokines, contributing to increased mucus production (**Figure 3**) (14). Type 2 inflammatory responses often manifest with heightened eosinophil counts or raised FeNO measurements (13). In contrast, non-type 2 inflammation involves the mechanism whereby macrophages engulf allergens like bacteria, viruses, and environmental toxins, subsequently stimulating T helper cells (TH cells). This stimulation results in the secretion of tumor necrosis factor α (TNFα) and interferon γ (INFγ). The release of these cytokines activates neutrophils, prompting them to produce agents such as matrix metalloproteinase 9 (MMP9), which facilitates the restructuring of the airways (3, 8).

When a patient is undergoing treatment with a high dose of ICS or taking OCS on a daily basis, the occurrence of any one of the following indicators may indicate the presence of type 2 inflammation: (1) A blood eosinophil count of ≥ 150 cells per microliter (μL); (2) FeNO ≥ 20 ppb; (3) Sputum eosinophils accounting for≥2% of the total cell count in the sputum sample; (4) Asthma that is clinically determined to be driven by allergens (3). Individuals requiring ongoing OCS might also exhibit type 2 inflammation. Ideally, these assessments ought to be performed prior to initiating OCS therapy, within one to two weeks following a course of OCS, or while the patient is on the minimal effective dose of OCS, because biomarkers indicative of type 2 inflammation are frequently diminished during OCS treatment (3).

Asthma is marked by the airways getting constricted, a consequence of ongoing inflammation in the airway linings. This inflammation involves immune cells crowding in and getting activated, which eventually leads to the airways getting blocked up because they’ve become narrower (15). The intricate interactions among various immune cells and nearby structural components, like epithelial cells, drive the emergence of asthma-related traits, including bronchial hyperresponsiveness (BHR). These manifestations are generally manageable and frequently reversible with bronchodilator therapy (15). In recent years, biologic therapies have been designed to address IL-4, IL-5, and IL-13, key components of type 2 inflammation, in severe asthma patients (16, 17). However, in certain severe cases of asthma, treatment doesn’t always fully restore normal airflow. For these individuals, persistent mucus blockages in the narrower airways could be the culprit behind the unchanging obstruction (18). Moreover, airway remodeling and other mechanisms may play a crucial role in disease development (15). Therefore, it is crucial to thoroughly understand which biological agents can alleviate airway obstruction and even reverse airway remodeling. Precise selection of biological agents for treating severe asthma patients is vital.

## 5 Biologic targeted therapy

Even when patients with chronic asthma diligently follow their high-dose ICS-LABA therapy and manage all relevant factors carefully, their condition often remains uncontrolled or worsens when the high-dose treatment is reduced. In such cases, it would be wise to consider the diagnosis of severe asthma (19). After confirming severe asthma, as mentioned earlier, most patients show a type 2 inflammatory phenotype. Therefore, it’s crucial to assess the phenotype before starting treatment (see **Figure 3**). The GINA 2024 report advises that biologic therapy should be reserved for individuals experiencing severe asthma, and should only be initiated after optimizing other treatments (3) (**Table 1**). The current types of biological agents mainly include anti-IgE, anti-IL4Rα, anti-IL5/5Rα, and anti-TSLP, among others. How to effectively choose suitable biological treatments for patients with severe asthma?

**Table 1:** Biologic targeted therapy for severe asthma patients with a phenotype of type 2 inflammation.

Features	Omalizumab	Mepolizumab	Benralizumab	Reslizumab	Dupilumab	Tezepelumab
Drug target	IgE	IL-5	IL-5Rɑ	IL-5	IL-4Rɑ	TSLP
Applicable age and population	≥6 years old; Allergic asthma, childhood onset,	≥6 years old; Eosinophilic asthma, adulthood onset	≥12 years old; Eosinophilic asthma, adulthood onset, polysorbate allergy	≥18 years old; Eosinophilic asthma, adulthood onset	≥6 years old;Eosinophilic or allergic asthma, childhood or adulthood onset	≥12years old;Eosinophilic or allergic asthma, adulthood onset, type 2 low asthma
Suitable for other diseases	nasal polyps; chronic spontaneous (idiopathic) urticaria	eosinophilic granulomatosis with polyangiitis(EGPA); hypereosinophilic syndrome; chronic rhinosinusitis with nasal polyps	unclear	unclear	moderate-to-severe atopic dermatitis; chronic rhinosinusitis with nasal polyps; eosinophilic esophagitis	unclear
FEV1	↑~↑↑	↑~↑↑	↑~↑↑	↑~↑↑	↑~↑↑	↑~↑↑
FeNO	↓	unclear	unclear	unclear	↓↓	↓↓
Blood eosinophils	↓	↓↓	↓↓	↓↓	unclear	↓
Sputum eosinophils	unclear	↓	↓	unclear	unclear	unclear
OCS	41% reduction in the proportion of patients receiving maintenance OCS	median OCS dose reduced by approximately 50%	median OCS dose reduced by approximately 50%	median OCS dose reduced by approximately 50%	median OCS dose reduced by approximately 50%	no effect
Clinical outcomes						
Exacerbations	↓↓	↓↓	↓↓	↓↓	↓↓	↓~↓↓↓
Quality of life	↑	↑	↑	↑	↑	↑
Adverse effects	Injection site reactions, anaphylaxis in approximately 0.2% patients (20).	In adults, injection site reactions, anaphylaxis rare; In children, headache, dizziness, syncope.	Injection site reactions; hypersensitivity reactions; helminth infection	Injection site reactions; hypersensitivity reactions; helminth infection;	Injection site reactions; hypersensitivity reactions; hypereosinophilia; conjunctivitis, helminth infection	Injection site reactions; hypersensitivity reactions; pharyngitis, arthralgia, back pain

OCS, Oral corticosteroid(s); mOCS, Maintenance oral corticosteroid(s);

↓: Implies a decline range of 0~30%; ↓↓: Implies a decline range of 31~60%; ↓↓↓: Implies a decline range of 61~100%.

↑: Increase range from 0 to 30%; ↑↑: Increase range from 31 to 60%; ↑↑↑: Increase range from 61 to 100%.

### 5.1 Anti-immunoglobulin E monoclonal antibody - *omalizumab*

Omalizumab, a specially designed monoclonal antibody, is used to fight the activity of IgE and has received approval for treating allergic asthma in children aged six and up. This treatment method specifically aims at the IgE pathway, which is pivotal in triggering allergic responses and worsening asthma symptoms. By attaching to IgE, omalizumab helps curb the release of inflammatory substances like histamine from mast cells and basophils, thus alleviating allergic reactions and enhancing asthma management. Clinical trials have confirmed its effectiveness in young patients, establishing it as a key option for tackling severe allergic asthma in the pediatric demographic (9). Furthermore, it is also authorized for addressing chronic spontaneous urticaria as well as chronic sinus inflammation accompanied by nasal polyps in both children and adults (21). Previous studies have demonstrated that omalizumab reduces asthma exacerbations and hospitalizations, while also providing modest enhancements in quality of life and pulmonary function (22). In a thorough review of multiple observational studies on people with acute allergic asthma, it was found that there was a significant 59% decrease in the frequency of exacerbations, a notable 41% decrease in the number of patients needing ongoing oral corticosteroid treatment, and a significant improvement in symptom management (23). Recent research indicates that omalizumab enhances the clinical results for patients suffering from severe asthma in real-world settings, demonstrating an effect size that closely parallels that observed in long-term randomized controlled trials (RCTs) (4). A practical study conducted by Wei Chern and colleagues revealed that individuals receiving treatment with omalizumab tend to be younger, show signs of disease onset earlier, and exhibit atopic characteristics. Additionally, these patients have a greater likelihood of having concurrent allergic bronchopulmonary aspergillosis and rhinitis (24), which seems to contradict the research by Susanne Hansen et al (25). The study by Susanne Hansen et al. have adopted a prospective cohort study design, the researchers could have followed 501 patients with severe asthma over one year, recording various aspects of their condition and treatment responses. On the contrary, the study by Wei Chern et al. is a retrospective study. The broader patient inclusion in the cohort study may capture a more diverse range of treatment responses, resulting in seemingly contradictory findings. Individuals who developed asthma in childhood and those with a medical history indicating allergen-induced symptoms show promising signs for a positive response to omalizumab (3).

Research on the efficacy of omalizumab for managing comorbid conditions associated with severe asthma remains sparse. However, two phase III randomized clinical trials led by Gevaert P et al. demonstrated that omalizumab significantly enhanced both subjective and objective nasal outcomes in patients suffering from nasal polyps (26). A recent research investigation revealed that children as young as one year old who suffer from multiple food allergies benefited more from a 16-week course of omalizumab compared to a placebo, as it significantly raised their tolerance to peanut and other widely recognized food allergens (21). Multiple studies indicate that omalizumab is effective for managing severe asthma in obese patients (27, 28). The 2024 GINA report highlighted various predictive factors linked to a favorable reaction to omalizumab for asthma treatment, as shown in **Table 2**.

**Table 2:** Potential predictors of good response to biological agents.

Biological agents	Potential predictors of good response
Omalizumab	1) Baseline IgE level does not predict likelihood of response (29); 2) Blood eosinophils ≥260/μL (30, 31) or FeNO ≥19.5 ppb (30); 3) Childhood-onset asthma (11); 4) Clinical history suggesting allergen-driven symptoms (11)
Mepolizumab Benralizumab Reslizumab	1) Higher blood eosinophils (strongly predictive) (32); 2) Higher number of severe exacerbations in previous year (strongly predictive) (11, 32); 3) Adult-onset asthma (33); 4) Nasal polyps (34); 5) Maintenance OCS at baseline (34); 6) Low lung function (FEV1 <65% predicted) in one study;
Dupilumab	1) Higher blood eosinophils (strongly predictive) (33); 2) Higher FeNO (strongly predictive) (33)
Tezepelumab	1) Higher blood eosinophils (strongly predictive) (11); 2) Higher FeNO levels (strongly predictive) (11).

### 5.2 Anti-interleukin-5 monoclonal antibodies-*mepolizumab* and *reslizumab*

IL-5 is primarily involved in the development of type 2-high severe asthma (8). The U.S. Food and Drug Administration (FDA) has approved mepolizumab and reslizumab, both interleukin-5 inhibitors, for the treatment of severe eosinophilic asthma (3, 35). Data from randomized clinical trials has shown that even with pre-existing conditions like upper respiratory issues, acid reflux, mental health challenges, diabetes, heart problems, or obesity, mepolizumab still manages to cut down on asthma flare-ups and boost asthma control, life quality and FEV1 in individuals facing severe eosinophilic asthma (36, 37). Indeed, within the group of patients diagnosed with severe asthma, the clinical improvements provided by mepolizumab were more significant in those suffering from CRSwNP, compared to those without this condition (38, 39). Unfortunately, the strict requirements for phase III clinical trials have limited the ability to apply these findings to larger, real-world populations. One study found that only 4% to 18% of people with severe asthma met the criteria to participate in trials testing new treatments (38). There is currently no research on the use of mepolizumab in the treatment of severe asthma complicated by obesity, food allergy, anaphylaxis, and allergic rhinitis, among other conditions.

Regarding the clinical efficacy of mepolizumab in the treatment of severe asthma patients, a study by Dennis Thomas et al. indicates that mepolizumab can induce remission in patients with severe asthma (40). A real-world investigation conducted by Wei Chern and colleagues revealed that individuals responding positively to mepolizumab tended to be older males who developed eosinophilic asthma later in life. This group also showed a greater tendency to have nasal polyps and, surprisingly, experienced fewer breathing difficulties, even though they were less prone to allergies (24), which was opposite to omalizumab. The 2024 GINA report indicated that certain factors may predict a positive response to anti-IL-5 therapy for asthma. These factors include elevated blood eosinophil levels, a history of more severe exacerbations in the past year, the use of maintenance oral corticosteroids at the outset, reduced lung function (FEV1 <65% of predicted), asthma onset in adulthood, and the existence of nasal polyps (3).

Reslizumab, the other anti-IL-5 drug that’s been given the thumbs-up, works by latching onto IL-5 as it circulates in the bloodstream, essentially preventing it from hooking up with the IL-5Rα receptor. Reslizumab got the green light back in 2017 for managing severe eosinophilic asthma in adults. Simone Hashimoto et. showed that reslizumab can really make a difference for people with severe eosinophilic asthma. It not only cuts down on how often they have serious asthma flare-ups, but it also helps them rely less on oral steroids. This held true whether they were just starting out with biologic therapy or were switching over from another type 2 biologic (41). A multi-center clinical trial showed that compared to the placebo group, the levels of eosinophils in the sputum and blood of the reslizumab group significantly decreased. Specifically, reslizumab reduced the level of eosinophils in sputum by an impressive 95.4%, and in blood by 38.7%. Moreover, only 8% of patients using reslizumab experienced worsening of their condition, while 19% of patients in the placebo group experienced a similar decline in their condition (42).

Patients with nasal polyposis receiving reslizumab showed some improvement in asthma control symptoms compared to those receiving a placebo. Furthermore, a couple of independent Phase 3 trials, encompassing 953 individuals with severe asthma and elevated blood eosinophil counts, demonstrated that reslizumab cut down on the yearly frequency of asthma flare-ups, boosted lung capacity, and generally provided better asthma control in those with severe eosinophilic asthma. However, these benefits weren’t apparent in patients whose baseline eosinophil levels were below 400 cells/μl (42, 43). Follow-up examination of the trial confirmed reslizumab’s efficacy and tolerability in individuals exhibiting refractory, advanced illness and high eosinophil counts (44, 45).

The activation of eosinophils in the airway mucosa is closely linked to bronchospasm, increased secretion of airway mucus, and, in some cases, structural changes in the air passages. Consequently, these cells are recognized as vital targets in the fight against asthma. While various cytokines and surface receptors contribute to the maintenance, proliferation, and activation of eosinophils, IL-5 and its corresponding receptors have garnered considerable focus from the scientific community. Research has demonstrated that IL-5 is instrumental in controlling the survival, migration, and activation of eosinophils (9, 46).

### 5.3 Anti-interleukin-5 receptor alpha monoclonal antibody - *benralizumab*

Benralizumab targets IL-5Rα on eosinophils and basophils (47). The FDA has given it the thumbs up for treating severe eosinophilic asthma in kids 12 and up. Over in Europe, though, the EMA has only signed off on it for adults (48). From landmark Phase 3 RCTs (SIROCCO, CALIMA), benralizumab demonstrably lowered exacerbations, enhanced pulmonary capacity, and lessened asthma impact (49, 50). Benralizumab has gotten the green light as an add-on maintenance therapy for those battling severe eosinophilic asthma that’s proving tough to manage. What’s more, the ZONDA trial that just wrapped up showed that throwing benralizumab into the mix helped cut down on the reliance on oral corticosteroids, got a better handle on asthma flare-ups, and didn’t mess with how much air patients could blow out in a second (FEV1), at least when stacked up against a placebo (51). It is of equal importance to note that benralizumab, when compared with alternative anti-IL5 therapeutic interventions, demonstrates an enhanced aptitude to reduce eosinophil counts with accelerated promptness and an approach towards comprehensive efficacy, inclusive of eosinophil-lineage committed progenitor cells present in both blood and sputum. This finding indicates a higher effectiveness in treating severe eosinophilic asthma, surpassing the efficacy of both mepolizumab and reslizumab (52, 53). Consequently, when a patient shows elevated levels of blood eosinophils, experiences a greater frequency of severe exacerbations in the past year, has developed asthma in adulthood, displays nasal polyps, and continues to rely on oral corticosteroids during the initial evaluation, it indicates a likely favorable outcome from anti-IL5 or anti-IL5Rα treatment options (see **Table 1**) (3, 45, 47).

### 5.4 Anti-interleukin-4 monoclonal antibody - *dupilumab*

Dupilumab was designed to target and bind to the alpha subunit of the IL-4 and IL-13 receptors, effectively suppressing the immunological responses triggered by these cytokines (54). A comprehensive meta-analysis of randomized controlled trials involved individuals suffering from severe asthma who had experienced at least one acute exacerbation within the previous year. The findings indicated that a treatment aimed at the anti-IL4Rα resulted in a 56% decrease in severe exacerbations. Furthermore, enhancements were noted in aspects such as quality of life, symptom control, and pulmonary function. While these improvements were statistically relevant, they fell short of achieving clinically significant levels (54–56).

In a retrospective evaluation, the clinical outcomes showed no difference between subjects with allergic and non-allergic phenotypes at the initial assessment (56). Individuals with severe asthma who rely on OCS saw a median 50% reduction in OCS dosage when treated with anti-IL4Rα therapy compared to a placebo. There were no strict requirements for peripheral blood eosinophil count or FeNO levels in this cohort (57). In individuals experiencing chronic rhinosinusitis accompanied by nasal polyps, dupilumab lessened polyp volume, alleviated nasal discomfort, and minimized reliance on systemic OCS or surgical intervention (58, 59). The Phase 3 LIBERTY ASTHMA VOYAGE trial set out to determine just how well dupilumab worked and how safe it was for kids aged 6 to 11 who were still struggling with moderate-to-severe type 2 asthma, even with their current treatment, over a year-long period. The results showed that dupilumab cut down on serious asthma flare-ups, helped them breathe easier, and generally got their asthma under better control, particularly in those with type 2 inflammation. As for safety, nothing popped up that we hadn’t already seen with dupilumab (60). Weight-tiered dosing regimens resulted in mean concentrations within the therapeutic range for dupilumab, with similar median decreases in levels of type 2 biomarkers across different dosing regimens (54). A phase 3 randomized, placebo-controlled study showed that dupilumab treatment resulted in a reduction in the annual frequency of severe asthma episodes and overall enhancement of lung function (61). On the whole, dupilumab was well-received in terms of safety within the QUEST study population (62). What’s more, the positive effects of the treatment were even more pronounced in patients who, at the start of the study, showed elevated levels of type 2 biomarkers (3).

### 5.5 Anti TSLP monoclonal antibody-*tezepelumab*

TSLP, a cytokine produced by epithelial cells, disrupts normal processes by activating a series of cell types and inflammatory pathways. This makes it a key factor in initiating and maintaining airway inflammation in asthma (17). When the epithelium encounters inhaled irritants such as allergens, viruses, or bacteria, it releases TSLP. Evidence suggests that TSLP kicks off a cascade of type 2 inflammation by activating a range of inflammatory cells and boosting type 2 cytokine production. Furthermore, lab experiments indicate that TSLP also has a hand in modulating certain facets of neutrophilic inflammation (17). TSLP levels in respiratory tract samples are elevated in asthmatics relative to controls, demonstrably correlating with both disease severity and compromised pulmonary capacity (63).

Tezepelumab is an antibody specifically designed to target TSLP. As the first biologic of its kind, it uniquely acts on cytokines derived from epithelial cells. This action helps to prevent TSLP from binding to its receptor, thereby reducing the immune response triggered by TSLP in various types of asthma (64, 65). In both the Phase 3 NAVIGATOR (66) and Phase 2b PATHWAY trials (65), tezepelumab really knocked it out of the park, substantially cutting down on asthma flare-ups compared to the placebo. This was true for patients wrestling with severe, uncontrolled asthma, no matter where their type2 inflammatory biomarker levels started. On top of that, it gave their lung function a boost, helped them get a better handle on their asthma, and improved their overall quality of life (67). In a randomized, placebo-controlled extension study, tezepelumab proved to be not only safe and sound over a two-year period, but also brought about enduring and significant reductions in asthma exacerbations for patients battling severe, refractory asthma (68). However, in patients already on maintenance oral corticosteroids, treatment with an anti-TSLP agent didn’t seem to help them lower their OCS dosage any more than a placebo did (3, 69). Early research and available findings suggest that specific biologics, such as dupilumab, mepolizumab, omalizumab, and tezepelumab, are effective in enhancing lung function, managing asthma, and reducing the frequency of asthma flare-ups in individuals with obesity-related asthma (70).

It appears that a diverse array of biologics is efficacy-laden in enhancing pulmonary function, mitigating exacerbation frequencies, and accomplishing favorable clinical outcomes for individuals afflicted with severe asthma. However, Pfeffer et al. observed anti-IL5/5R’s clinical advantage in lessening asthma flare-ups and ICS dependence (71). Individuals with compromised lung function or those facing the possibility of rapid deterioration might gain from prompt intervention, particularly if elevated baseline levels of BEC and FeNO, either individually or together, are indicative of potential improvements in lung function tied to biological factors (72). Consequently, it is imperative to initially categorize the patient before selecting a biological agent for the treatment of an individual with severe asthma, subsequently opting for an appropriate biologic therapy contingent upon the prospective predictive factors indicative of a favorable response.

## 6 Histopathological effects

It’s common knowledge that the hallmark pathological features of asthma stem from a tangled web of immune cell infiltration and interaction, which in turn causes ongoing inflammation of the airway walls (8, 73). In severe asthma, this constant inflammation can really do a number on the bronchial walls, leading to structural changes we call remodeling (74, 75). These changes go hand-in-hand with ongoing inflammation, showing up as increased growth of the bronchial wall. Airway remodeling has a few key characteristics, most notably an increase in the number of mucosal glands and goblet cells, a thickened basement membrane, the growth of fibroblasts and myofibroblasts in the submucosa, enlargement of the airway smooth muscle, and the development of new blood vessels, which is known as neovascularization (76, 77).

Contemporary research extensively highlights the critical involvement of IgE and its binding sites in disease development (78). In their empirical study, Roth et. elucidated that stimulation of bronchial smooth muscle by IgE leads to the excessive synthesis of types I, III, and VII collagens and fibronectin, which are all key components of the extracellular matrix (79). In the annals of 2012, the investigation conducted by Riccio Am et. elucidated that a considerable segment of individuals grappling with severe asthma experienced a marked decrement in the baseline bronchial reticular basement membrane (RBM) thickness, as well as a diminished eosinophilic infiltration, subsequent to a 12-month therapeutic regimen with omalizumab. This finding underscores the potential therapeutic capacity of omalizumab to modulate the airway remodeling process in those beset by severe, persistent allergic asthma (80). Makoto Hoshino and his collaborators orchestrated an investigation utilizing computed tomography (CT) scanning to ascertain the impact of omalizumab on the caliber of airway wall thickness. The findings elucidated that omalizumab was instrumental in diminishing both the thickness of the airway walls and the inflammatory responses within the airways (81) (**Table 3**). There is burgeoning evidence suggesting that omalizumab has the potential to diminish the basement membrane’s thickness and alleviate fibronectin deposits within the airways of individuals afflicted with asthma. Moreover, it serves as a preventive measure against inflammation triggered by exacerbations, thereby maintaining the integrity of the airway lining (**Table 3**) (76).

**Table 3:** The study of the pathological changes in severe asthma patients treated with biological agents.

Study (first author, publication year)	Study population	Biological agents	Methods for evaluating results	Effects on pathological changes of severe asthma
Christian Domingo (2023) (77)	31	Omalizumab	Primary efficacy outcome :(a) change in OC monthly dose by the end of treatment. Secondary efficacy outcomes :(a) spirometry changes, (b) airway inflammation [fraction exhaled of nitric oxide (FeNO)], (c) number of exacerbations and (d)reversibility of the histological changes in the bronchial mucosa	Facilitate the repair of the bronchial epithelium, given that some of the patients returned to normality.
A.N. Frix (2020) (81)	157	Omalizumab	ACQ,AQLQ,FeNO, Sputum and blood eosinophils and neutrophils FEV1 Exacerbations	Reducing exacerbation rate, improving patient perspective outcomes and airway calibre, together with reducing type-2 airway inflammation.
Weronika Zastrzeżyńska (2020) (75)	13	Omalizumab	Inhaled corticosteroid dose ACQ,AQLQ, FEV1 Exacerbations Blood eosinophilia Blood monocytes Oral corticosteroid dose Basal lamina thicknessFibronectin deposit in airway mucosa	Decrease unfavorable airway structural changes in allergic asthmatics, at least with respect to the fibronectin deposit and increased thickness of the basal lamina.
Michael Roth (2015) (82)	40	Omalizumab	Primary efficacy outcome includes (a) change in OC monthly dose by the end of treatment. Secondary efficacy outcomes include: (a) spirometry changes, (b) airway inflammation (FeNO) (c) number of exacerbations and (d) reversibility of the histological changes in the bronchial mucosa.	Showed a marked OC-sparing capacity and was associated with an improvement in clinical management that correlated with bronchial epithelial repair.
Pierluigi Mauri (2014) (29)	8	Omalizumab	Reticular basement membrane (RBM) thickness Protein profiles	Down-regulated bronchial smooth muscle proteins in severe asthma.
Makoto Hoshino (2012) (80)	30	Omalizumab	Serum total IgE, Morning PEF, FEV1, ACQ, AQLQ, Sputum eosinophils	Reduced airway wall thickness and airway inflammation.
Riccio AM (2012) (79)	11	Omalizumab	RBM thickness Bronchial eosinophils	Reduced the original bronchial RBM thickness and eosinophil infiltration
Laura Bergantini (2023) (83)	34	Mepolizumab	ACQ-6, ICS dose, OCS dose Eosinophils Treg cells T effector cells	Anti-IL-5 treatment induces a rebalancing of Treg and T effector cells in patients with severe asthma.
Patrick Flood-Page (2003) (84)	24	Mepolizumab	Bronchial mucosal eosinophils Reticular basement membrane: tenascin, procollagen III, and lumican, FEV1 Peak expiratory flow rate (PEFR) Histamine PC20	Decrease in expression of ECM proteins in the airway RBM. Decrease in TGF-β1 expression by airway eosinophils
Tomoko Tajiri (2024) (85)	28	Dupilumab	Cough and sputum symptoms Radiological mucus scores on CT Airway dimensions on CT Annualized rate of asthma exacerbations FeNO and pulmonary function ACQ-7 AQLQ	Dupilumab could reverse subjective and objective measures of airway mucus hypersecretion and airway remodeling in adult patients with uncontrolled moderate-to-severe asthma.
Nicola A. Hanania, MD, MS (2023) (86)	1902	Dupilumab	FEV1 FEV1/FVC ACQ-5 AQLQ Blood eosinophils FeNO	In patients with uncontrolled moderate-to-severe asthma, treatment with dupilumab facilitates reversal of persistent airflow obstruction status and improves clinical outcomes.
Sarah Diver (2021) (66)	250	Tezepelumab	Oral corticosteroid use; Use of maintenance treatments in addition to inhaled corticosteroids FEV1, ACQ-6, Exacerbations, FeNO Blood eosinophil count, Serum total IgE, Perennial specific IgE status, Mean serum IL-5 Mean serum IL-13, Reticular basement membrane thickness, Airway epithelial integrity	Reduced airway eosinophil counts regardless of baseline blood eosinophil count Reduced airway hyperresponsiveness to mannitol
Adatia A (2023) (87)	39	Tezepelumab	FEV1, FVC, PD15 ACQ56 FeNO IgE Eosinophils Sputum eosinophils Sputum neutrophils IL-33 levels in BALF	Reduced the airway epithelial inflammatory response including IL-33 and T2 cytokines to viral challenge without affecting anti-viral host resistance. Stabilizes the bronchial epithelial immune response to respiratory viruses.
Andreasson LM (2024) (88)	229	Tezepelumab	Eosinophilic status, FEV1, FeNO AHR (PD15 to mannitol), Skin prick test IgE, Blood eosinophils Sputum eosinophils, Sputum neutrophils ICSs,Eosinophilic status BAL eosinophils	TSLP in sputum was associated with the degree of AHR in patients with asthma irrespective of eosinophil levels supporting the role of AHR.

Moreover, omalizumab has proven to be highly effective in managing severe asthma when assessed in real-world scenarios. Its ability to reduce the frequency of exacerbations, improve overall patient outcomes, and increase airway openness, along with its impact on reducing type-2 airway inflammation, underscores its significant value in treatment (84) (**Table 1**). The research suggests that omalizumab has the potential to undo the complex airway remodeling that happens in severe asthmatics, and mepolizumab seems to be just as effective. In a groundbreaking study, Flood-Page et. analyzed the bronchial biopsies of people with mild atopic asthma who were only using short-acting beta-agonists (SABAs). They looked closely at the changes in the tissue before and after the subjects received three doses of mepolizumab (89, 90). Studies indicate that individuals with mild asthma exhibit a marked increase in both the quality and density of tenascin within the respiratory basal membrane (RBM), alongside a rise in the count of eosinophils that are positive for transforming growth factor-β1 (TGF-β1+). When compared to their healthy counterparts, these asthmatic individuals show elevated levels of TGF-β1 in the bronchoalveolar lavage (BAL) fluid. Notably, the administration of mepolizumab not only leads to a reduction in eosinophils in the bronchi but also diminishes the presence of TGF-β1+ eosinophils, lessens the thickness of the basal membrane, reduces tenascin immunoreactivity, and lowers the TGF-β1 concentration in the BAL fluid (89). Only one clinical study (a 12-month trial comparing mepolizumab to a placebo, involving 61 subjects) has looked into how mepolizumab affects airway remodeling (AR). After adjusting for body surface area, the CT scans revealed a noteworthy difference: the mepolizumab group’s average shift in both the wall and total areas was significantly greater than that of the placebo group. In plain English, the mepolizumab group saw a decrease in these values, whereas the placebo group experienced an uptick (75, 91). In a similar vein, Cachi et al. dug into how benralizumab affects airway remodeling in severe asthma by taking a look at patient biopsies (83, 90). Compared to the placebo, benralizumab knocked down the number of eosinophils hanging out in the bronchial lamina propria, as well as the airway smooth muscle (ASM) mass. Interestingly, among those getting benralizumab, the proliferation of myofibroblasts didn’t seem to budge when stacked up against the control group. As for how benralizumab trims down ASM mass, it looks like it’s happening indirectly, mainly by wiping out the local transforming growth factor-β1-positive (TGF-β1+) eosinophils chilling within the bronchial lamina propria (90). Moreover, Laura Bergantini and her colleagues demonstrated that therapeutic intervention with anti-IL-5 elicits a restorative equilibrium between regulatory T cells and effector T cells within the pulmonary microenvironment of individuals afflicted with severe asthma (86). Additional comprehensive inquiries are requisite to ascertain the probable function of reslizumab in the context of the mechanisms involved in the pathogenesis of airway remodeling in individuals afflicted with asthma (75).

Dupilumab, as previously indicated, is a fully human monoclonal antibody that effectively impedes the common receptor constituent for interleukins IL-4 and IL-13. These cytokines serve as pivotal and fundamental mediators of type 2 inflammation across a spectrum of pathologies (92). IL-4 elicits the activation of mast cells, orchestrates the differentiation of T helper cells towards the type 2 phenotype, and drives class switching to IgE in B lymphocytes. At the same time, IL-13 throws fuel on the fire of airway remodeling by spurring the proliferation of smooth muscle cells, causing goblet cells to multiply, and revving up mucus production. It also gives fibroblasts a nudge to pump out more extracellular matrix proteins, which leads to a thickening of the subepithelial basal membrane (93, 94). All of this, apart from AHR, is classic airway remodeling (94). Therefore, dupilumab is projected to affect both excessive airway mucus production and airway structural alterations (85). After 48 weeks of treatment with dupilumab, Tomoko Tajiri and colleagues’ observational study found that the CASA-Q’s four cough and sputum scores all saw considerable gains. Moreover, imaging studies showed a marked decrease in mucus accumulation and a lessening of airway wall thickening as seen on CT scans. These drops in mucus levels correlated significantly with improvements in overall AQLQ scores, reduced airway obstruction, and a decline in type 2 inflammation within the airways (92, 95). Hanania and his team’s research demonstrates that dupilumab administration can turn the tide for patients grappling with moderate to severe, poorly controlled asthma. The drug not only alleviates chronic airflow obstruction but also leads to a marked improvement in clinical outcomes across the board (96).

AHR is a crucial pathophysiological characteristic of asthma, linked to heightened contractility of the airway smooth muscle stemming from the infiltration of mast cells and eosinophilic inflammation in the airways (67). The cytokine TSLP prompts a transformation in airway mast cells, resulting in an increase of a chymotrypsin-positive phenotype observed in asthmatic individuals experiencing AHR, particularly those with severe and uncontrolled asthma. Consequently, inhibiting TSLP could offer a promising strategy for mitigating AHR (88). The erudite research team of Andreasson LM (97) and colleagues has harnessed the precision of an ultrasensitive assay to ascertain the presence of TSLP within serum, sputum, and bronchoalveolar lavage specimens. Notably, TSLP concentrations within sputum have been correlated with the magnitude of airway hyperresponsiveness (AHR) in asthmatic patients, regardless of eosinophil count, thereby affirming the significance of AHR and TSLP as indicants of the malady that warrant further investigation in both eosinophil-rich and eosinophil-poor asthma subsets (97). Moreover, TSLP has been identified as a possible factor in airway remodeling due to its role in promoting collagen synthesis by fibroblasts and the growth of airway smooth muscle (87). *In vivo* studies using tezepelumab to inhibit TSLP in asthma patients showed a decrease in the airway epithelial inflammatory response, including reductions in IL-33 and type 2 cytokines when faced with a viral challenge, all while preserving the body’s natural resistance to viruses. Findings from Sverrild A et al. indicate that inhibiting TSLP helps to stabilize the immune response of the bronchial epithelium against respiratory viruses (20).

## 7 Conclusion and future directions

### 7.1 Clinical efficacy insights

The results of this review clearly demonstrate that biological therapies have shown remarkable potential in managing severe asthma patients with a Type 2 inflammation phenotype. Clinically, these therapies have led to significant improvements in multiple aspects. By reducing the number of exacerbations, biological therapies not only enhance the patient’s daily functionality but also potentially decrease the burden on healthcare systems associated with emergency room visits and hospitalizations (39, 58). Furthermore, patients have experienced an improvement in lung function parameters (48). Measures such as FEV1 and peak expiratory flow (PEF) have shown positive trends, indicating enhanced airway patency and better respiratory function. The enhancement in lung capacity leads to a noticeable decrease in symptoms, including breathlessness, wheezing, and coughing, enabling patients to participate in their everyday activities with much more comfort. In addition to physiological improvements, biological therapies have also had a positive impact on patients’ quality of life. Asthma often restricts patients’ physical activities, social interactions, and sleep quality (19). The alleviation of symptoms and improvement in lung function have led to an overall enhancement in patients’ well - being, enabling them to participate more fully in their lives and reducing the psychological burden associated with the disease.

### 7.2 Pathological histology discoveries

From a pathological histology perspective, our review reveals that biological therapies can effectively modulate the underlying type 2 inflammatory processes. There is evidence of a decrease in the infiltration of eosinophils, a key cell type associated with type 2 inflammation in asthma. This reduction in eosinophil numbers indicates a suppression of the eosinophilic - mediated inflammatory cascade, which is known to contribute to airway remodeling, mucus hypersecretion, and bronchoconstriction. Moreover, changes in other histological markers related to type 2 inflammation, such as levels of cytokines and chemokines, have been observed. Biological therapies seem to be able to regulate the production and release of these inflammatory mediators, thereby dampening the overall inflammatory response in the airways. This modulation of the inflammatory microenvironment is crucial for preventing long - term airway damage and the progression of asthma.

### 7.3 Implications for clinical practice

Based on these findings, biological therapies should be seriously considered as an integral part of the treatment algorithm for severe asthma patients with a type 2 inflammation phenotype. Clinicians need to be vigilant in identifying patients with this specific phenotype, as early initiation of biological therapy can potentially lead to better outcomes. This requires a comprehensive approach to patient assessment, including the measurement of biomarkers such as blood eosinophil counts, FeNO levels, and other type 2 - related biomarkers.

Once a patient is identified as suitable for biological therapy, close monitoring is essential. Regular assessment of clinical symptoms, lung function, and biomarker levels can help in determining the effectiveness of the treatment and making any necessary adjustments. Additionally, patient education is of utmost importance. Patients need to be informed about the nature of biological therapies, their potential benefits, and possible side effects to ensure compliance and active participation in their treatment.

### 7.4 Future research directions

Despite the significant progress shown in this review, there are still several areas that warrant further investigation. Firstly, more long - term studies are needed to assess the durability of the effects of biological therapies. While short - to medium - term efficacy has been demonstrated, understanding how these therapies perform over extended periods, and whether there are any long - term side effects or development of resistance, is crucial.

Secondly, research into the optimal patient selection criteria is still ongoing. Although type 2 inflammation biomarkers have been used to identify potential responders, there may be other factors, genetic or otherwise, that could further refine the selection process. Identifying these additional factors could help in tailoring treatment more precisely, ensuring that patients most likely to benefit receive the appropriate therapy. Finally, studies comparing different biological therapies directly in well - designed randomized controlled trials are necessary. This would provide more definitive evidence on the relative efficacy, safety, and cost - effectiveness of different agents, enabling clinicians to make more informed treatment decisions.

In conclusion, biological therapies for severe asthma with a type 2 inflammation phenotype have shown great promise in both clinical and pathological histology terms. However, continued research and refinement of treatment strategies are essential to fully realize their potential and improve the lives of patients suffering from this debilitating condition.
